# Toward Diverse Plant Proteins for Food Innovation

**DOI:** 10.1002/advs.202408150

**Published:** 2024-08-09

**Authors:** Woojeong Kim, Canice Chun‐Yin Yiu, Yong Wang, Weibiao Zhou, Cordelia Selomulya

**Affiliations:** ^1^ School of Chemical Engineering UNSW Sydney NSW 2052 Australia; ^2^ Department of Food Science and Technology National University of Singapore Singapore 117542 Singapore

**Keywords:** food application, functional properties, plant protein, protein interaction, protein structure

## Abstract

This review highlights the development of plant proteins from a wide variety of sources, as most of the research and development efforts to date have been limited to a few sources including soy, chickpea, wheat, and pea. The native structure of plant proteins during production and their impact on food colloids including emulsions, foams, and gels are considered in relation to their fundamental properties, while highlighting the recent developments in the production and processing technologies with regard to their impacts on the molecular properties and aggregation of the proteins. The ability to quantify structural, morphological, and rheological properties can provide a better understanding of the roles of plant proteins in food systems. The applications of plant proteins as dairy and meat alternatives are discussed from the perspective of food structure formation. Future directions on the processing of plant proteins and potential applications are outlined to encourage the generation of more diverse plant‐based products.

## Graphical abstract

Plant proteins are high‐quality materials to form food structure and to replace meat and dairy counterparts. This comprehensive review outlines molecular structure, processing, and application of plant proteins from diverse biological sources highlighting their techno‐functional properties. Their structure in food systems, fundamental properties, and future directions are also provided offering guidance for assorted plant‐based product production.

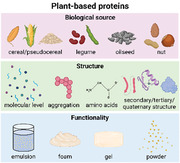

## 1 Introduction

With the impact of the current consumption pattern of animal products, the push for plant‐based food is motivated by environmental, health, and ethical concerns. Current industrial animal farming practices are widely regarded as unsustainable contributing to 12% of total anthropogenic greenhouse gas (GHG) emissions at 6.2 billion tons of CO_2_ equivalent (GtCO_2_eq) in 2015. This figure is predicted to increase by 46.8% to 9.1 GtCO_2_eq by 2050, assuming no change to habits and intervention.^[^ ^1^ ^]^ The contribution by animal‐based food (57%) is almost double that of plant‐based food production (29%) in the total GHG emissions by food production.^[^ ^2^ ^]^ Moreover, animal farming is often associated with extensive land use and the loss of habitat.^[^ ^3^ ^]^ The land use per 1000 calories for animal meat stands between 6.61m^2^ (poultry) and 119.49 m^2^ (beef), in contrast with the minimal land use of peas (2.16 m^2^), nuts (2.11 m^2^), and rice (0.76 m^2^).^[^ ^4^ ^]^ As arable land is under threat in a changing climate, a multitude of strategies must be employed to ensure the food security of future generations.^[^ ^5^ ^]^ To this end, plant‐based foods could be a sustainable solution to reduce the reliance on animals as a protein source.^[^ ^5^, ^6^ ^]^

The last decade saw the commercialization of plant‐based food analogs.^[^ ^7^ ^]^ Efforts to improve the quality of specific plant‐based food analogs have proposed possible formulations for meat, fat, cheese, and milk analogs that may be comparable to their animal‐based counterparts. In plant‐based meat, the focus was placed on mimicking the mouthfeel and texture of meat, as the replication of the fibrous texture of meat remains challenging.^[^ ^8^ ^]^ For products like plant‐based cheese, increasing efforts were directed to provide a solution with superior melt‐stretch characteristics to current products.^[^ ^9^ ^]^ Functional design of meat and dairy alternatives with plant proteins has led to the creation of food ingredients‐based structures such as emulsions, foams, and gels.

The structure and interactions of food proteins that form colloidal building blocks impact the techno‐functional properties. As plant and animal proteins play different roles, remarkable differences to animal proteins in primary, secondary, and tertiary structures are presented in plant proteins.^[^ ^10^ ^]^ The functionality of plant proteins is often marred by low solubility in water, and weak or even inability to stabilize emulsions and form gels.^[^ ^11^, ^12^, ^13^ ^]^ For instance, prolamins are compact plant storage proteins that are insoluble in water due to proline and glutamine residues.^[^ ^10^ ^]^ This stands in contrast to the random nature of casein and whey protein, which allow high solubility and flexibility.^[^ ^10^ ^]^ The processing and modification of plant proteins to alter their properties have underpinned the improved functionality.^[^ ^14^, ^15^ ^]^ Simple heat treatments were traditionally used to achieve higher functionality of plant proteins, while a wide range of physical and chemical techniques demonstrated effectiveness in inducing structural changes in protein, including ultrasonication, high pressure treatment, extrusion, and enzymatic treatment.^[^ ^11^ ^]^

Unlike animal sources, a wide variety of plant sources are available as a potential protein supply.^[^ ^16^ ^]^ To date, several plant sources that have been exploited and used for protein supplements include soybean,^[^ ^17^ ^]^ pea,^[^ ^18^ ^]^ chickpea,^[^ ^19^ ^]^ wheat,^[^ ^20^ ^]^ maize,^[^ ^21^ ^]^ buckwheat,^[^ ^22^ ^]^ rapeseed,^[^ ^23^ ^]^ flaxseed,^[^ ^24^ ^]^ and sunflower.^[^ ^25^ ^]^ Aside from the usual commodities, further explorations into different regional and ethnic food sources were also seen such as amaranth,^[^ ^26^ ^]^ sorghum^[^ ^27^ ^]^ and quinoa.^[^ ^28^ ^]^ Food researchers have traditionally investigated the structure and properties of established plant proteins such as soy, pea, wheat, and rice, while research on functional properties of lupin, fava bean, maize, barley, and canola have been recently emphasized to form effective food structure.^[^ ^29^, ^30^ ^]^ Waste valorization has come up as a new trend for obtaining plant‐based proteins.^[^ ^31^ ^]^ The major byproducts from food manufacture are soy whey, soybean pulp, meals and cakes of cereals and oilseeds composed of valuable components including proteins, polysaccharides, phenolic compounds, etc.^[^ ^32^ ^]^ The residual cakes are known to contain up to 50% protein content depending on the type of wastes.^[^ ^33^ ^]^ The advancement of purification techniques has enabled efficient isolation of these proteins from byproducts for food applications.^[^ ^34^ ^]^

Despite extensive research on the processing of plant proteins in recent decades, there remains a lack of comprehensive information on molecular structure of plant proteins from various sources during production and their impacts on structure formation in food colloids. Existing literatures only focused on a single aspect of modification methods, improving the functionality, extraction technologies of plant proteins, and simple comparison with animal proteins.^[^ ^35^, ^36^, ^37^, ^38^, ^39^, ^40^, ^41^ ^]^ Reflecting the latest trends of processing technologies of plant proteins, it is necessary to understand the relationship between protein structure at molecular and particle levels and macroscopic properties and behavior in food colloids, leading to design effective plant‐based products. Therefore, this review discusses the native structure of plant proteins from a wide variety of botanical sources and recent development of processing technologies in various food colloids including emulsions, foams, and gels. The ability to characterize structural, morphological, and rheological properties highlighting advanced biophysical analytical tools and their applications in food systems were also outlined to assess the performance and potential to substitute dairy and meat counterparts.

## 2 Production of Plant‐Based Proteins

Production of plant proteins has a significant impact on protein quality including purity, composition, and structure, which influences its structure formation in food formulations. This section discusses molecular structure and composition of plant proteins subject to the factors affecting the protein production and soluble/insoluble protein aggregation during processing before the use as food ingredients.

### 2.1 Molecular Structure and Composition of Plant Proteins upon Extraction

A variety of plant proteins used as food ingredients are derived from cereal, pseudocereal, legumes, oilseeds, and others, and their molecular structures vary depending on the source.^[^ ^42^ ^]^ Seed storage proteins are classified simply into four types: water‐soluble albumin; salt‐soluble globulin; prolamin soluble in alcohol/water mixture; and glutelin soluble in acidic or alkaline solutions.^[^ ^43^ ^]^ Albumin includes 2s‐types such as leucine, while globulin includes 7s vicilin and 11s legumin in peas, soy, and rice. Prolamin includes gliadin, zein, hordein, and secalin from wheat, maize, barley, and rye, while glutelin includes glutenin in wheat.^[^ ^18^, ^44^, ^45^, ^46^ ^]^

The native colloidal state of plant proteins is highly related to cultivars, and their aggregation would be driven under the influence of extraction conditions such as pH, solvent, salt, and temperature, resulting in the coexistence of the native state and aggregates of the proteins.^[^ ^47^, ^48^, ^49^, ^50^, ^51^ ^]^ The cultivar significantly impacts the structure and composition of plant proteins. Pea protein isolates from various cultivars prepared by alkaline extraction‐isoelectric precipitation were found to exhibit differences in solubility and emulsifying properties due to differences in the contents of legumin, aggregates, sulfhydryl group, and β‐turn structure depending on cultivars.^[^ ^49^ ^]^

**Figure 1:**
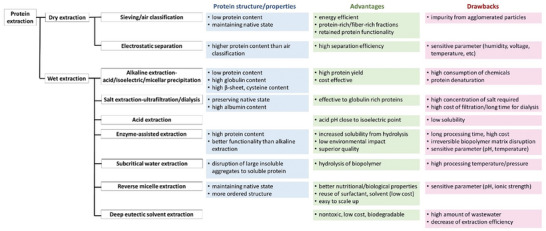
Dry and wet protein extraction technologies with protein structure/properties, advantages, and drawbacks.

The extraction methods have a more significant influence on the protein content, composition, and structure rather than the cultivars.^[^ ^52^ ^]^ Protein structure/properties, advantages, and drawbacks of dry and wet protein extraction technologies are listed in **Figure** **1**. Moreover, recent extraction technologies to assist cell disruption include microwave, ultrasound, pulse electric field, and high hydrostatic pressure‐assisted extractions, although more research is needed to understand the changes in protein composition and structure.

Dry extraction of plant proteins from cereal and pulses could pose more advantages in energy efficiency and maintenance of native protein structure compared to wet extraction.^[^ ^53^, ^54^, ^55^, ^56^ ^]^ Generally, dry fractionation starts with milling, where spherical particles with starch granules and protein bodies are found.^[^ ^56^, ^57^ ^]^ Next, air classification is used to remove starch and obtain fine powder with small particle size and high protein content with increasing wheel speed of the classifier.^[^ ^55^ ^]^ Changes in the amino acid composition of yellow pea, mung bean, and cowpea were observed after dry fractionation, reporting that dry fractionation did not affect amino acid composition in all proteins, while yellow pea shows a lower hydrophobic: hydrophilic amino acid ratio than mung bean and cowpea, suggesting that protein composition is more dependent on the protein source.^[^ ^56^ ^]^ Although studies on dry fractionation of plant proteins mainly focus on increasing protein content due to high starch content and low protein purity (50%–60%), further data on structure, composition, and morphology are still needed.

In dry extraction, no contact is needed between protein and water, thus the proteins could maintain their native state despite low protein content (≈60%), while wet extraction can lead to structural rearrangements of monomers and oligomers.^[^ ^53^, ^54^, ^58^ ^]^ In a typical wet extraction, rehydration and precipitation steps affect the protein conformation.^[^ ^59^, ^60^, ^61^ ^]^ The precipitation step alters protein composition because several albumin fractions remain soluble during isoelectric precipitation and micellar precipitation.^[^ ^59^ ^]^ Extraction methods using alkaline/salt extraction‐ultrafiltration and salt extraction‐dialysis, instead of precipitation, retain a relatively high content of albumin, while relatively high globulin content was retained in alkaline extraction‐isoelectric precipitation and micellar precipitation.^[^ ^61^ ^]^ The rehydration step also significantly affects protein structure. In the case of rehydration with an alkaline solution, the 11s/7s ratio was higher as more hydrophobic sites were exposed, resulting in higher protein denaturation than when a salt solution was used.^[^ ^62^ ^]^ Relatively higher contents of β‐sheet in protein secondary structure and cysteine were found using an alkaline solution compared to a salt solution.^[^ ^60^ ^]^

Among extraction technologies particularly for plant proteins, reverse micelle technology is an emerging alternative method to preserve the native structure of a protein during extraction.^[^ ^63^ ^]^ The drawbacks of two‐way wet extraction, alkaline extraction‐isoelectric precipitation (AEIP), include high water demand, high consumption of chemicals, and protein denaturation from pH shifting. In contrast, reverse micelles are formed by surfactants that are dispersed in organic solvent where the extraction process is divided into two steps: forward extraction and backward extraction. Proteins are solubilized into aqueous cores of the reverse micelles in the forward extraction, and by adding an aqueous solution, the solubilized proteins in the reverse micelles are separated to aqueous phase by passing an interfacial layer through protein‐surfactant interactions.^[^ ^64^ ^]^ Several studies have compared plant protein quality extracted using reverse micelle technology to AEIP.^[^ ^65^, ^66^ ^]^ More ordered structure (higher α‐helix and lower random coil contents) was found in reverse micelle‐extracted protein than typical wet‐extracted proteins, suggesting reverse micelle preserved the original structure of proteins with a higher amount of free sulfhydryl content, while alkaline extraction caused the degradation of protein molecules with lower free sulfhydryl content due to alkali treatment.^[^ ^65^, ^66^ ^]^

Deep eutectic solvent (DES) extraction for plant proteins have arisen as a promising method due to low toxicity, biodegradability, and sustainability.^[^ ^67^ ^]^ The soluble aggregate formation when dispersing into the DES allows proteins to be distributed, which does not destroy the protein structure.^[^ ^68^ ^]^ Choline chloride‐based DES with different hydrogen bond donor exhibited high extraction efficiency, while retaining the tertiary structure of plant proteins.^[^ ^68^ ^]^ To date, various plant protein including soy,^[^ ^69^ ^]^ rapeseed^[^ ^70^ ^]^ oat,^[^ ^71^, ^72^ ^]^ fava bean,^[^ ^73^ ^]^ sesame^[^ ^74^ ^]^ have been produced using DES within a recent decade. While DES method exhibit low extraction efficiency due to high viscosity and poor selectivity of the proteins, the extraction process is still being developed considering the factors of processing temperature,^[^ ^70^ ^]^ solvent composition^[^ ^71^, ^72^, ^73^ ^]^ to minimize protein denaturation and to obtain proteins with high purity. Especially, the development of DES system using synthesis with butanediol, dihydric alcohol and mixing with glycerol, ethylene glycol, etc. is highlighted.^[^ ^71^, ^73^, ^74^ ^]^ Recent attempts on ultrasound‐assisted DES extraction showed potential to produce high‐quality proteins from Sacha inchi meal biomass^[^ ^75^ ^]^ and microalgal biomass^[^ ^76^ ^]^ by breaking down the cell walls from ultrasound treatment.

### 2.2 Molecular Structure and Particle Agglomeration of Plant‐Based Powders

Dry extraction and drying methods of plant protein colloids are key factors influencing the composition and structure of plant protein powder. More importantly, drying including freeze drying and spray drying greatly impacts powder properties. Freeze drying typically leads to larger particle sizes with high protein content, while spray drying represents better functionality.^[^ ^77^, ^78^ ^]^ Spray drying results in more disulfide bonds with an amorphous and less ordered structure, while freeze drying shows a more ordered structure.^[^ ^79^, ^80^ ^]^

Particle agglomeration is the aggregation of particles using binders to form larger porous secondary particles affecting rehydration in food powders, and this behavior is much more known for dairy proteins compared to plant proteins.^[^ ^81^, ^82^, ^83^, ^84^ ^]^ For example, whey protein with high protein content and poor wettability shows enhanced wettability and rehydration time with agglomeration.^[^ ^82^, ^83^ ^]^ On the other hand, because agglomeration only modifies external structure, it is difficult to accelerate the dispersion process with agglomeration in the case of casein with its micellar structure.^[^ ^82^, ^83^ ^]^

Agglomeration of plant proteins typically improves the flowability and wettability of powders.^[^ ^85^, ^86^, ^87^, ^88^ ^]^ Agglomerated particles formed by soy protein with carboxymethyl cellulose as a binder exhibited a more porous and irregular shape with an increase in particle size to 130% of its original size.^[^ ^85^ ^]^ Subsequently, increasing binder concentration was found to form a larger and stronger granule, while increasing the binding feed rate reduced the growth rate in forming rice protein agglomerates using konjac glucomannan as a binder.^[^ ^87^ ^]^ A strong solid bridge between particles is formed with higher binder concentration to avoid breaking the granule, while air temperature is the most important in granule size because agglomeration can only occur when the particles are sufficiently wet.^[^ ^86^ ^]^ The flowability of agglomerated particles in rice and pea proteins was much higher than that of the raw material.^[^ ^86^, ^87^ ^]^

### 2.3 Soluble Aggregate Formation of Plant Proteins

**Figure 2:**
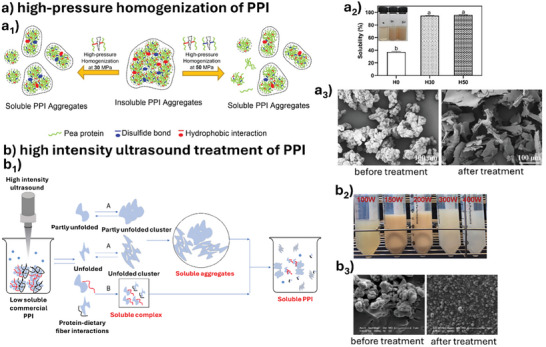
Physicochemical treatment of pea protein isolate (PPI) to form soluble aggregates: a) high pressure homogenization; a_1_) a schematic illustration to form soluble protein aggregates from insoluble protein aggregates, a_2_) solubility of PPI dispersions subject to pressure, a_3_) scanning electron microscopic images of PPI dispersions before/after treatment (Reproduced with permission.^[^ ^96^, ^97^ ^]^ Copyright 2022, Elsevier and Copyright 2022, Elsevier). b) high‐intensity ultrasound treatment; b_1_) proposed mechanisms for the formation of soluble protein aggregates by high intensity ultrasound treatment, b_2_) macroscopic images of PPI dispersions subject to ultrasound intensity, b_3_) scanning electron microscopic images of PPI dispersions before/after treatment (Reproduced with permission/under terms of the CC‐BY license.^[^ ^99^, ^100^ ^]^ Copyright 2016, Elsevier and Copyright 2022, Elsevier).

Commercial plant proteins are often spray dried upon extraction. Protein concentration and the subsequent drying can induce protein aggregation, resulting in large insoluble particle formation when dissolved in water and/or other solvents.^[^ ^89^, ^90^ ^]^ The solubility for commercial plant proteins is generally lower than 30%, and therefore physical/mechanical treatments, especially high pressure homogenization, and high‐intensity ultrasound treatment, are often needed to form soluble aggregates (**Figure** **2**).^[^ ^91^, ^92^ ^]^ Thus, this section discusses soluble aggregate formation of plant proteins with processing to improve solubility in water before use.

High pressure jet processing of commercial pea and soy protein isolates enhanced their structure and physicochemical properties in aqueous dispersions.^[^ ^93^ ^]^ High pressure processing improves protein solubility by separating large protein clusters^[^ ^94^, ^95^ ^]^ (Figure 2a). Commercial plant proteins such as pea protein, are a cluster of aggregates, whereas high pressure homogenization breaks down the aggregates into an amorphous and flake‐like structure.^[^ ^96^ ^]^ As the pressure increased, the particle size decreased, and solubility increased.^[^ ^93^, ^97^ ^]^ High pressure homogenization with 30–50 MPa was found to break down non‐covalent and covalent bonds (disulfide bonds) of pea protein isolates.^[^ ^97^ ^]^ However, the generation of monomeric protein units was not possible even at higher pressures (up to 500 MPa).^[^ ^93^ ^]^

High‐intensity ultrasound treatment is another way to produce soluble aggregates of commercial plant proteins (Figure 2b). The cavitation effect during ultrasound treatment exposes hydrophilic amino acid groups by disrupting hydrogen and hydrophobic interactions, thereby increasing the surface area of the protein and solubility.^[^ ^98^ ^]^ With increasing intensity, the trends of increase in β‐sheet and the decrease in α‐helix and β‐turn were observed.^[^ ^99^ ^]^ The large aggregates disappeared, and less dense, looser, and smaller clusters were more prominent as the ultrasound intensity increased.^[^ ^99^ ^]^ The changes in particle shape due to ultrasound treatment are generally more significant in plant proteins than animal proteins. O'Sullivan et al.^[^ ^100^ ^]^ compared the structural changes of animal and plant proteins subjected to ultrasound treatment, indicating a structural change of soy protein isolate to monodisperse and spherical particles from large aggregates, while bovine gelatin with a fibrous structure formed fibrils with similar width and shorter length of a single fiber. Nevertheless, no change was found in the primary structure of molecular weight profiles for both proteins.

Heat or acid‐induced protein aggregates are typically applied to form structures in emulsions, foams, and gels. For example, soluble aggregates of soy protein isolate are formed by heat treatment due to denaturation of vicilin and convicilin fractions with partial insoluble macroaggregates upon heating above 90 °C.^[^ ^101^ ^]^ Other processing methods such as enzymatic cross‐linking,^[^ ^102^, ^103^ ^]^ coacervation,^[^ ^104^, ^105^ ^]^ and physical separation^[^ ^106^ ^]^ have been developed to form soluble plant protein aggregates, and they affect the size, dispersibility, structure, and functionality of protein aggregates.^[^ ^107^ ^]^ The choice of protein is important when applying these methods as the outcomes may vary. In the case of coacervation, a smaller size of mung bean protein coacervates with better re‐dispersibility was generated compared to those of soy protein due to additional preparation steps including heating, drying, and redispersion of protein colloids.^[^ ^108^ ^]^ Enzyme‐mediated cross‐linking was attempted for fava bean protein and oat protein, where transglutaminase has superior cross‐linkability for plant proteins compared to tyrosine, while tyrosine reduced the solubility of a particular protein (oat in this case) and showed limited effect on fava bean protein.^[^ ^109^ ^]^ This is because glutamines in oat and fava bean proteins are abundant, and transglutaminase could cross‐link between glutamine and lysine residues, whereas tyrosine binds tyrosine residues with other tyrosine, lysine, or cysteine.^[^ ^109^ ^]^ Pea protein aggregation promoted by thermodynamic incompatibility in the pea protein‐sodium alginate aqueous system showed larger and denser particle formation with increasing alginate proportion.^[^ ^106^ ^]^

The use of plant proteins to stabilize Pickering emulsions has been thoroughly covered in a review written by Sarkar & Dickinson.^[^ ^110^ ^]^ Plant proteins such as zein and gliadin that are not easily soluble in water were utilized to form Pickering particles by solvent‐driven methods. Details are described in Section 2.4 as Pickering particles are mainly used to stabilize the oil/water interface.

## 3 The Role of Plant Proteins in Food Colloids

### 3.1 Plant Proteins in Emulsions

#### 3.1.1 Bulk Emulsion

**Figure 3:**
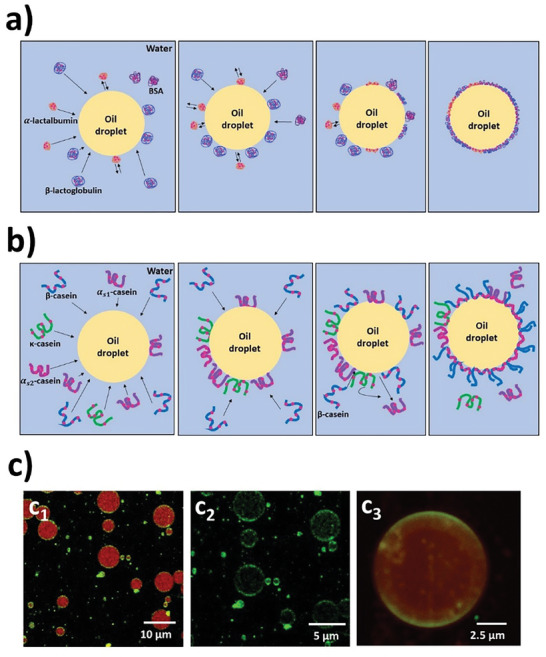
Emulsifying properties of dairy proteins. a) Emulsifying mechanisms of whey protein (Reproduced with permission.^[^ ^111^ ^]^ Copyright 2020, Elsevier), b) emulsifying mechanisms of casein (Reproduced with permission.^[^ ^111^ ^]^ Copyright 2020, Elsevier), c) confocal images of whey protein isolate‐stabilized emulsions (unpublished data); red color indicates oil droplets and green color indicates the protein interfacial layer.

Food proteins as natural emulsifiers are of particular interest owing to their amphiphilicity by nature.^[^ ^111^ ^]^ Dairy proteins have been widely used as protein‐based emulsifiers because milk itself is a natural form of oil‐in‐water emulsion.^[^ ^112^ ^]^ Emulsifying mechanisms of dairy proteins including whey protein and casein at the molecular level are described in **Figure** **3**. Whey protein with monomeric globular structure and casein with flexible structure are preferential to strongly adsorb at the oil/water interface and form an interfacial layer (Figure 3a,b). A thin and viscoelastic interfacial layer of proteins surrounding oil droplets is usually seen (Figure 3c).

**Figure 4:**
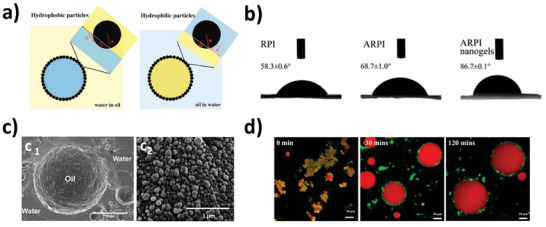
Emulsifying properties of plant protein Pickering particles. a) Three‐phase contact angle subject to emulsion type (Reproduced with permission.^[^ ^111^ ^]^ Copyright 2020, Elsevier), b) contact angles of rapeseed protein nanogel with treatments (Reproduced under the terms of the CC‐BY license.^[^ ^115^ ^]^ Copyright 2020, ACS Publications), c) cryo‐SEM image of plant protein microgel‐stabilized emulsion (c_1_, Reproduced under the terms of the CC‐BY license.^[^ ^116^ ^]^ Copyright 2020, ACS Publications) and soy protein‐anthocyanin complex nanoparticle‐stabilized Pickering emulsion (c_2_, Reproduced with permission.^[^ ^117^ ^]^ Copyright 2020, Elsevier), d) confocal image of plant protein microgel‐stabilized Pickering emulsion as a function of in vitro gastric digestion time (Reproduced under the terms of the CC‐BY license.^[^ ^116^ ^]^ Copyright 2021, ACS Publications).

Nevertheless, increasing use of plant proteins as food emulsifiers is anticipated, due to the recent demand for plant‐based foods and clean labels in the food and beverage sector. Comparisons between plant proteins and animal proteins in emulsions showed that animal proteins including whey protein, egg protein, and gelatin show superior functionality compared to some plant proteins such as pea and potato proteins due to the high solubility of animal proteins.^[^ ^113^, ^114^ ^]^ Therefore, increasing the solubility of plant proteins is imperative to improve their roles as emulsifiers.^[^ ^89^ ^]^ Furthermore, plant proteins often exist in the form of complex globular structures, and/or aggregates, and thus their emulsifying abilities could be understood at a particle level. Emulsifying properties of plant protein Pickering particles are summarized in **Figure** **4**.

Pickering emulsion utilizes solid particles as a stabilizer, starting with zein among plant proteins due to low solubility in water and oil, while the method to produce the Pickering particles has been expanded to use other plant‐based sources including prolamin‐rich cereals and globular legumes.^[^ ^111^ ^]^ The contact angle is one of the most important factors in the design of Pickering particles since effective Pickering particles, being partially wet in both water and oil phases due to the capillary forces, possess a contact angle close to 90**°** (Figure 4a).^[^ ^118^ ^]^ The anti‐solvent method developed to produce zein Pickering particles has been further implemented to other prolamin‐rich plant proteins such as karfirin in sorghum,^[^ ^119^ ^]^ hordein and secalin in barley,^[^ ^120^ ^]^ and gliadin in wheat.^[^ ^121^, ^122^ ^]^ Pickering particles in the form of nanoparticles or microgels have also been investigated using complex globular soy, pea, and peanut proteins by pH adjustment, thermal treatment, and dissolution.^[^ ^123^, ^124^, ^125^, ^126^, ^127^ ^]^ Recently, nanogels prepared by self‐assembly of acylated rapeseed protein produced a near 90**°** of contact angle (86.7**°**), suggesting the suitability to stabilize Pickering emulsions (Figure 4b).^[^ ^115^ ^]^ Moreover, Pickering emulsions stabilized by plant proteins display the capability for controlled release with the unique particle structure upon digestion, in which large droplets are covered by Pickering particles with pepsin addition (Figure 4d).^[^ ^116^ ^]^

Although Pickering particles form a single layer at the oil/water interface, some particles act as structuring agents with other substances such as polysaccharides, forming high internal phase Pickering emulsions (HIPPE).^[^ ^128^ ^]^ Plant proteins are in favor of forming HIPPE with viscoelastic and self‐standing features.^[^ ^125^, ^129^ ^]^ HIPPE stabilized by plant protein particles is attracting attention due to its superior biocompatibility, tunable physicochemical and rheological properties, and cost‐competitive advantage, while their performance in commercial food products and in vitro and in vivo studies should be further evaluated.^[^ ^128^ ^]^

Research and development on plant/dairy protein blends have been accelerated to compensate for the poor functionality of plant proteins. Several reviews on the synergism of plant/dairy protein blends and strategies to enhance their techno‐functionality have been provided.^[^ ^111^, ^130^, ^131^, ^132^ ^]^ Kim et al.^[^ ^111^ ^]^ focused on the emulsifying mechanisms of plant/dairy protein blends, emphasizing different adsorption behaviors and interactions between proteins depending on the type of dairy and plant proteins. For example, whey‐legume protein blends and legume protein blends show enhanced emulsion stability, while sodium caseinate‐legume protein blends formed physically unstable emulsions.^[^ ^133^ ^]^ Protein structure and type are closely related to the competitive absorption of heteroproteins at the oil/water interface, which will be covered in Section 3.1.2.

#### 3.1.2 Protein Adsorption at Oil/Water Interface

Understanding the absorption behavior and interfacial properties of proteins at the oil/water interface is essential for stable emulsion production.^[^ ^134^ ^]^ Protein absorption behavior at the oil/water depends on the subphase because hydrophobic interaction exists in the oil phase.^[^ ^135^ ^]^ Soy protein isolate shows greater affinity at the tetracane‐water interface than the air/water interface.^[^ ^136^ ^]^ Similarly, plant proteins such as peas, soy, mung bean, and rice formed more elastic interfacial films when using triglyceride and terpene as subphases than air/water interface due to faster structural rearrangement at the oil/water interface.^[^ ^137^ ^]^ In particular, the surface pressure is the lowest when using triglyceride (12 mN m^−1^ for triglyceride and 22 mN m^−1^ for terpene), consistent with the fact that surface energy is readily reduced when using oil with lower polarity.^[^ ^135^, ^137^ ^]^

Protein molecules fundamentally approach the oil/water interface and undergo structural rearrangement to form an interfacial layer by interacting with other protein molecules. However, the stabilization mechanism of plant proteins at the oil/water interface highly depends on protein composition. Plant globulin, representatively 7S globulin (also known as β‐conglycinin and vicilin, 150–190 kDa) and 11S globulin (also known as glycinin and legumin, 300–380 kDa), which most commonly accounts for 90% of soy and 50%–65% pea proteins, show different behavior at oil/water interface.^[^ ^138^ ^]^ Legumin with a relatively large molecular weight is slow to diffuse to the interface, but they are stable once attached, while vicilin with a relatively small molecular weight readily attaches to the interface and tends to go back and forth.^[^ ^111^, ^139^, ^140^ ^]^ Legumin shows higher interfacial affordability and only a small amount of vicilin is found at the interface due to competitive adsorption in soy protein‐stabilized emulsions.^[^ ^141^ ^]^

The botanical source of protein affects the interfacial properties due to their composition. Legume protein including soy and pea proteins show similar tendency when absorbed, whereas rice protein shows lower elasticity with weaker interaction between proteins at the oil/water interface, forming a weak interfacial layer.^[^ ^137^ ^]^ Legume proteins including soy and pea proteins primarily consist of globulins and albumins, while rice protein mainly contains prolamin and glutelin. When comparing the interfacial properties of brown rice protein, hemp protein, and soy protein, soy protein formed the most solid‐like interfacial film with low consistency, while brown rice formed a less structured interface with higher viscosity.^[^ ^142^ ^]^ In addition, hemp protein was found to form a viscoelastic interfacial layer with intermediate mechanical properties to soy and brown rice proteins and high compatibility with sunflower oil, leading to improved emulsion stability.

Plant protein forms a less stiff and more ductile interfacial layer, whereas animal protein forms a solid and viscoelastic layer.^[^ ^139^ ^]^ The molecular conformation of plant proteins is more affected by pH than animal proteins, where soy protein can form an interface with viscoelasticity with a good absorption rate at pH lower than 5 or high alkaline conditions for example.^[^ ^143^ ^]^ Among plant proteins, legume proteins including soy and pea proteins tend to form a thick and elastic layer at the interface.^[^ ^142^, ^144^, ^145^ ^]^ Except for soy and pea proteins, wheat gliadin with high surface activity, flexible structure, and transformability is promising for forming a viscoelastic interfacial layer than some other sources (corn zein or sunflower).^[^ ^144^, ^146^, ^147^ ^]^ Nevertheless, the interfacial properties of plant proteins need to be improved for practical application. Physicochemical treatments including thermal treatment, pH shifting, enzyme, and chemical modification result in protein unfolding, making them flexible to form a viscoelastic interfacial layer, while more research is required on protein adsorption and interfacial properties of modified plant proteins.^[^ ^148^, ^149^, ^150^ ^]^

Research on stabilizing the oil/water interface using plant/dairy protein blends is emerging to complement the shortcomings of plant proteins. However, due to competitive absorption between dairy and plant proteins, one protein tends to be displaced by another protein, causing emulsion destabilization.^[^ ^151^, ^152^ ^]^ For example, in the pea/whey protein blends, whey protein replaces pea protein over time at the oil/water interface.^[^ ^151^, ^153^ ^]^ In addition, the interfacial properties could be controlled according to the order of incorporating proteins.^[^ ^132^, ^154^ ^]^ Compared to sole protein, the simultaneous addition of pea and whey proteins could form a more viscous interface, resulting in high flocculation and low stability, while sequential adsorption could control the emulsion properties including droplet size and the degree of flocculation.^[^ ^154^ ^]^ Adding pea protein to pre‐absorbed whey protein could form a stable emulsion with a thick interfacial layer, whereas adding whey protein to pea protein or simultaneous addition may cause the replacement of pea protein fractions with β‐lactoglobulin.^[^ ^132^ ^]^

A few strategies have been provided to avoid the competitive absorption of heteroproteins.^[^ ^152^, ^155^ ^]^ Adding polysaccharides with high viscosity is found to prevent the pea protein fractions from being displaced by whey protein fractions.^[^ ^152^ ^]^ Enzymatic cross‐linking between heteroproteins was also suggested as a strategy to stabilize the oil/water interface by modifying protein structure with increased β‐sheet and surface hydrophobicity, making the proteins more amphiphilic.^[^ ^155^, ^156^ ^]^ This topic possesses potential with numerous combinations of proteins, especially not only the animal/plant protein blends, but also the blends between plant proteins, and their processing should be further investigated.

### 3.2 Plant Protein‐Stabilized Foams

#### 3.2.1 Plant Proteins as Foam Stabilizers

Food foams formed by gases in liquids are found in a variety of application in food industry including whipped cream, ice cream, cakes, bread, and mousses. Egg albumin (egg white) has been dominating the research on food foams with its meringue‐forming properties, while overall understanding of foaming systems is still not properly investigated. Early studies on modifying whey proteins were targeted to mimic the foaming properties and stability of egg albumin, resulting in cost‐effective products.^[^ ^157^, ^158^ ^]^ Nevertheless, the recent drive of many studies is to utilize plant proteins as a substitute of egg proteins with the demand from consumers for more plant‐based alternatives.

Since egg albumin was the main source generating stable foams, the use of only albumin fractions from plant proteins is emerging, rather than by applying excessive processing for protein structure modification.^[^ ^159^, ^160^, ^161^ ^]^ Globulin fractions generally form foams with poor stability due to their aggregated structure forming a weak interfacial film, whereas albumin fraction showed compatible foam stability to whey protein due to cohesive interfacial layer formation around air bubbles when using proteins from mung bean, Bambara groundnut, and yellow pea.^[^ ^161^ ^]^ Subsequently, mild extraction of Bambara groundnut proteins was found to lead to higher foam stability than extensive purification because more albumins are retained from mild purification.^[^ ^159^ ^]^ The potential use of pea albumins was evaluated subject to pH and heat treatment, suggesting the albumin fractions were most stable at pH 3, by not forming aggregates during heat treatment.^[^ ^162^ ^]^ However, the molecular properties of albumins from diverse plant sources need to be understood before applying them as foam stabilizers.

Pickering foams are known to have a longer stability than conventional foams by forming tightly‐packed interfacial layer from adsorbed protein particles for preventing bubble shrinkage and gas diffusion and/or forming gel‐like network in the aqueous phase for reducing drainage and depletion stabilization.^[^ ^163^ ^]^ While zein is well known to stabilize Pickering emulsions, Qu et al. (2024)^[^ ^164^ ^]^ recently attempted the zein Pickering foams by inducing deamidation with alcohol‐soluble sugar derivatives and applying typical antisolvent precipitation. Overall, sugar derivatives (sucralose, maltitol, mannitol, stevioside) tended to improve the foamability and foam stability due to improved flexibility and hydrophilic aggregation of zein, but stevioside led to optimum foamability and foam stability driven by van der Waals forces and hydrogen bonds. Soy protein microgels fabricated by either heat‐induced or enzyme cross‐linking methods demonstrated that enzyme‐induced microgel stabilized foams via Pickering‐type mechanism with aggregated gel‐like structure at air/water interface, showing improved long‐term stability.^[^ ^165^ ^]^

#### 3.2.2 Processing Technologies to Improve Foamability and Foam Stability of Plant Proteins

Physicochemical treatments including cold plasma treatment, ultrasound, pH shifting, homogenization, heat treatment, microwave treatment, etc. have been applied to improve foaming properties of various plant proteins including aquafaba, pea, chickpea, and rice glutelin. Regardless of the treatment, the foamability of legume proteins (pea and chickpea) improved by reducing the protein aggregates. For example, short‐term cold plasma treatment of pea protein led to better foamability due to the reduction of intramolecular aggregates. Moreover, the combined treatment of chickpea protein (ultrasound and pH shifting) and rice glutelin (heat treatment and pH shifting) improved the foamability due to the reduced aggregate size and structural flexibility.^[^ ^166^, ^167^ ^]^ However, pH was found to affect foam stability and surface dilatational properties in potato proteins, not surface activity and foamability,^[^ ^168^ ^]^ suggesting ultrasound and/or heat treatment may contribute more to adjusting surface activity of the proteins that reduces interfacial tension. Incorporation of small/large molecules to plant proteins have addressed the limitation of the foaming capacity and stability in plant proteins.

Interactions of plant proteins with polysaccharides and polyphenols induced by enzymatic cross‐linking^[^ ^169^ ^]^ and complexation at various pH.^[^ ^170^, ^171^ ^]^ Incorporation of polysaccharide tended to improve the foam stability by altering the structure of plant proteins and/or forming a secondary layer at air/water interface after plant proteins primarily adsorbed at the air/water interface.^[^ ^172^ ^]^ The structural modification of plant proteins by interacting with polyphenols according to physicochemical processing technologies have been recently reviewed demonstrating that polyphenol incorporation to plant protein generally improve the foaming properties by reducing gas bubble size due to the increase in random coil content and molecular flexibility to stabilize air/water interface.^[^ ^173^ ^]^ The authors also noted that the foaming properties increase with increasing polyphenol content, while an excessive amount of polyphenol could lead to antagonistic effect by promoting protein aggregation.

### 3.3 Gelation of Plant Proteins

Plant‐based food gels have been an essential element of many traditional cuisines. In creating food gels, both plant‐based proteins and polysaccharides may be manipulated into forming a 3D structure with texture beyond their original form. Until recently, most studies have focused on common plant‐protein sources such as soy, pea and gluten.^[^ ^174^ ^]^ Exploration into alternative protein sources such as peanuts,^[^ ^175^ ^]^ and mung bean^[^ ^176^ ^]^ revealed opportunities in novel food design. Although plant protein is capable of forming gels alone, protein‐polysaccharides mixed gels were also made to enhance existing and create unique properties.^[^ ^177^ ^]^ Understanding the interactions between composites is instrumental in unlocking possible uses of plant protein in food.

**Figure 5:**
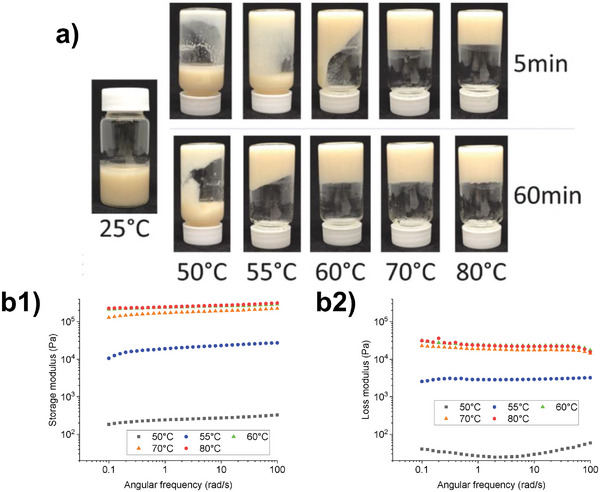
Empirical definition of gels. a) Inverted tubes of pea protein and curdlan gel after 5 and 60 minutes of heating. Successful gelation is indicated by the lack of flow of the sample upon inversion. b) Small amplitude oscillatory strain frequency sweep measured at 25 °C of pea protein and curdlan gel formed by different temperatures. (Adapted with permission.^[^ ^180^ ^]^ Copyright 2023, Elsevier).

Food gels may be defined qualitatively through the tube inversion method, where the gel does not flow toward the direction of gravity when its container is inverted^[^ ^178^ ^]^ (**Figure** **5**a). In research, gels were routinely defined qualitatively through their rheology, defined by the storage modulus (G′) being greater than its loss modulus (G″) over an extended oscillatory frequency range. A small amplitude oscillatory shear (SAOS) frequency sweep of a pea protein and curdlan gel set at different temperatures can be seen in Figure 5b. The difference in the moduli's respective values indicates the gel strength of the loaded sample, where G″ is at least 1 magnitude greater than the G′, the gel is regarded as strong and vice versa.^[^ ^179^ ^]^

**Figure 6:**
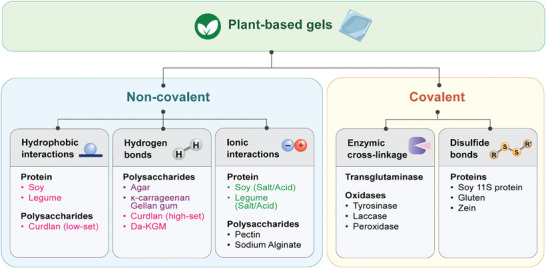
General schematics for food gels by the type of interactions with examples of molecules where the gel is created by said mechanism. Orange: heat‐induced gelation; Blue: cold‐set gelation; Yellow: salt/acid‐induced gelation; Grey: salt‐induced gelation.

Types of common food gels may be largely categorized by their gelation mechanism as seen in **Figure** **6**. Both physical and chemical gels are seen in food. Physical gels are a product of non‐covalent interactions (hydrogen bonds, electrostatic interactions, hydrophobic interactions) between molecules that are induced by heat or the addition of salts. Chemical protein gels that are covalently cross‐linked are also present in food through enzymatic‐induced gelation and the formation of disulfide bonds. Since gel properties are determined by their molecular structure and gelation condition, this section aims to divulge the effects of using various plant materials and their processing conditions.

#### 3.3.1 Gelation Mechanism and Molecular Structures

Non‐covalent interactions: Since food hydrocolloids are complex organic structures, various properties of plant hydrocolloids have been exploited to form a continuous structure. Generally, the gelation of native plant hydrocolloids would involve heat treatment and/or the addition of coagulants such as salts and acids.

**Figure 7:**
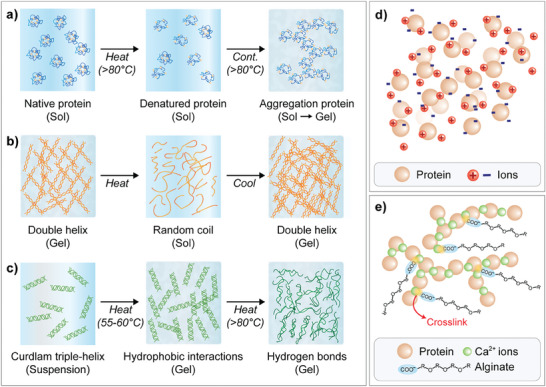
Illustrations of selected gels formed by non‐covalent interactions between food molecules. a) Heat‐induced protein gelation. b) Cold‐set gelation of food polysaccharides. c) Morphology of low‐ and high‐set curdlan gel. d) Aggregation of protein molecules by the reduction of electrostatic repulsion in salt‐/acid‐induced gelation. e) Alginate‐protein composite gel crosslinked by calcium ion.

Heat treatment is one of the most common methods employed for gelation in full or as a step to assist and improve the action of added coagulants. In plant protein systems, high temperatures are typically used to aggregate and agglomerate protein molecules via the exposure of hydrophobic sites. Above the gelation onset temperature, proteins begin forming aggregates which would subsequently result in a continuous network as aggregated protein agglomerates.^[^ ^181^ ^]^ An illustration of the heat‐induced gelation process can be seen in **Figure** **7**a. This mechanism applies to legumes such as soy,^[^ ^182^ ^]^ yellow pea, lentil, and fava bean proteins, where the globulins form the continuous structure in legume‐based gels.^[^ ^183^ ^]^ Although peanut protein is often regarded as having poor gelling properties, a recent study showed that the properties of heat‐set peanut protein gel may be improved by pH‐shifting pre‐treatment.^[^ ^184^ ^]^ When peanut protein isolate was subjected to alkaline pre‐treatment at pH10, gels strength and WHC were enhanced compared to the untreated protein samples. The effect was attributed to changes in the secondary and tertiary structures increasing free sulfhydryl groups and surface hydrophobic sites. Improvement in gelling properties of underutilized protein via processing is instrumental to be able to diversify plant protein sources.

In protein gel systems involving polysaccharides, heat plays several roles in the creation of food gels. Certain natural cold‐set polysaccharides like agar, κ‐carrageenan (κC), and gellan gum require heat to solubilize before gelation via cooling.^[^ ^185^, ^186^, ^187^ ^]^ At high temperatures, these polysaccharides are dispersed in an aqueous solution as random coils.^[^ ^188^ ^]^ Gelation occurs when the solution temperature decreases to their respective sol‐gel transition temperatures. Hydrogen bond interactions between polysaccharide coils initiate the formation of double helices that will be aggregated into a 3‐D structure upon cooling^[^ ^185^ ^]^ (Figure 7b). In these systems, the addition of cold‐set polysaccharides enables a greater latitude of design in thermal behavior for products like dairy dessert mimetics.^[^ ^189^ ^]^ Furthermore, the addition of κC was also shown to improve the structure of soy protein isolate (SPI) gel. The interaction between SPI and κC was electrostatic and hydrogen by nature and was most predominantly found at the ratio of 95:5 (SPI: κC).^[^ ^190^ ^]^ At this ratio, κC and SPI were found to form complex coacervates resulting in a stiffer gel (G″_max_ 3.5 Pa to G″_max_ 36.8 Pa).^[^ ^190^ ^]^

Conversely, heat‐set gelation relies on elevated temperatures to induce gelation. Aside from native and certain modified starches, emerging interest was seen in using other heat‐set polysaccharides in conjunction with plant protein in novel food development. Curdlan gum (CUD) may form both thermal‐reversible gel (low‐set, 55–60 °C) and irreversible gels (high‐set, >80 °C).^[^ ^191^ ^]^ At high‐set temperatures, the disassociation of the CUD triple helix led to a homogenous gel with enhanced hydrogen and hydrophobic interactions.^[^ ^192^ ^]^ A schematic of curdlan gelation may be seen in Figure 7c. This disassociation of the curdlan triple helix was observed to entangle protein molecules in a soy protein gel, forming a denser and more uniform microstructure, which manifested in WHC improvements.^[^ ^193^ ^]^

On the other hand, gelation of both plant protein and polysaccharide may be induced or assisted by the addition of a salt‐based or acidic coagulant (Figure 7d). The addition of salt/acid promotes the aggregation of protein by neutralizing the electrostatic repulsion through its ionic strength and lowering the pH to the isoelectric point respectively.^[^ ^194^, ^195^ ^]^ Typically, the use of a coagulant will be preceded by a heating step to form soluble protein aggregates by exposing charged sites of protein molecules.^[^ ^196^ ^]^ When salt is added, a salt bridge is formed by the cation across negatively charged regions in the proteins forming a continuous stranded gel microstructure.^[^ ^197^, ^198^ ^]^ The salt type has a direct effect on gel properties such as rheology,^[^ ^199^ ^]^ texture,^[^ ^199^, ^200^ ^]^ taste,^[^ ^200^ ^]^ and water‐holding capacity (WHC)^[^ ^201^ ^]^ that is dependent on the salt's coagulation power,^[^ ^202^ ^]^ their respective concentration,^[^ ^203^ ^]^ and solubility in water.^[^ ^204^ ^]^

Acidification of the protein solution can induce gelation by both the addition of organic acids^[^ ^205^ ^]^ (glucono‐𝛿‐lactone (GDL), citric acid, ethanoic acid) and through fermentation.^[^ ^206^ ^]^ The disassociation and release of the proton ion from acidification neutralizes the electrostatic repulsion between protein molecules, resulting in the aggregation of protein to form a 3‐D gel.^[^ ^207^ ^]^ Protein aggregation was reported to start near the isoelectric point of the protein due to differences in charge for protein fractions.^[^ ^208^ ^]^ Acid‐induced protein gels were reported to create a more homogenous microstructure than salt‐induced gels.^[^ ^201^ ^]^ Nonetheless, the selection of an acidifier is also critical to the final gel properties. Cao et al.^[^ ^205^ ^]^ reported that a slower rate of acidification favored the formation of a stronger gel. These same observations were corroborated by Yang et al.^[^ ^209^ ^]^ where a slow lactic acid fermentation produced a stronger gel than both GDL and citric acid.

Composite gel with interdependent networks may also be formed between proteins and polysaccharides via a salt cross‐linking agent. The gelation of polysaccharides like alginates is associated with the “egg‐box” model where ionic cross‐linkages are formed at junction zones between the cation and the disassociated carboxylic acid groups (R‐COO^−^) on adjacent polysaccharides, constructing a pseudo‐semiflexible network^[^ ^179^, ^210^ ^]^ (Figure 7e). Given the non‐specific binding nature of these cations, interlinking at the charged site and junction between the protein and alginate network was hypothesized, resulting in superior gel strength.^[^ ^211^ ^]^ Synergism between polysaccharide and protein also improved its extrudability by shifting molecular structure and creating interpenetrating network.^[^ ^212^, ^213^ ^]^ Oyinloye and Yoon^[^ ^214^ ^]^ demonstrated that pea protein‐alginate composite gel was suitable for food 3D printing as it exhibited excellent extrusion behavior with high viscosity before complete gelation at 43 °C. All mixed network was shown to have increased textural quality compared to alginate‐only gels. Notably, an 80:20 ratio of alginate to pea protein was optimal for ease of extrusion and mechanical strength. Thus, advancement in composite gels formulation enables the inclusion of proteins to generate foods with complex forms such as intramuscular fat in steak‐like meat analogs.

Covalent interaction: In food gels, the formation of covalently bound polymer “true” gels is less common than the above‐described physical gels. In food applications, the creation of chemically bonded gels typically involves enzymatic treatment or the formation of disulfide bonds in certain proteins.^[^ ^215^ ^]^

**Figure 8:**
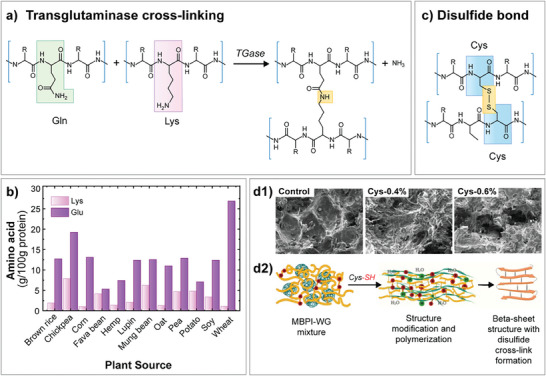
Covalent cross‐linking of protein gels. a) TGase cross‐linking of glutamine (Gln) and lysine (Lys) residues in protein (Red box). b) Lysine and glutamic acid (Glu) content in various plant protein isolates in literature.^[^ ^229^, ^230^, ^231^, ^232^ ^]^ Glutamic acid was reported as a proxy for glutamine content due to acid hydrolysis.^[^ ^229^ ^]^ c) Formation of a disulfide bond between two cystine (Cys) residues in protein (Green box). d1) Extracted SEM micrograph at 0%, 0.4%, and 0.6% L‐cysteine content within a mung bean protein isolate and gluten matrix. d2) Illustration of the roles of L‐cysteine within the gluten and mung bean protein isolate.

Enzymatic cross‐linkage of protein can induce cold‐set gelation that negates high‐temperature processing during gelation. Transglutaminase (TGase) is a typical enzyme for protein gelation, nonetheless, other enzymes like tyrosinase (Tyr) and laccase were also frequently used^[^ ^216^ ^]^ to induce gelation in plant proteins such as soy,^[^ ^199^, ^217^ ^]^ pea,^[^ ^218^ ^]^ zein,^[^ ^219^ ^]^ and potato.^[^ ^220^ ^]^ Interestingly, cross‐linkage between polysaccharides or between proteins and polysaccharides was shown to be possible in cases where oxidase enzymes were used.^[^ ^221^ ^]^ Isopeptide bonds are formed by TGase catalysis, the acyl‐transfer reaction between peptide‐bounded glutamine (Glu) and lysine (Lys) residues at their respective carboxyamine and ϵ‐amino groups, resulting in a 3D gel structure.^[^ ^176^, ^222^ ^]^ **Figure** **8**a shows the chemical linkage between the groups and the comparative content of glutamic acid and Lys in common plant protein sources. On the other hand, tyrosinase and laccase are both copper‐containing oxidases that oxidize a wide range of substrates into quinones that would cross‐link with other phenolic molecules.^[^ ^223^, ^224^ ^]^ For tyrosinase, a two‐step enzymatic reaction generates quinones by oxidation of monophenols like that in tyrosine that could cross‐link with other protein sidechains (lysyl, tyrosyl, cysteinyl, and histidinyl).^[^ ^216^ ^]^ Contrastingly, laccase catalyzes the one‐step formation of quinones free radicals through the removal of one electron that when in excess could polymerize with surrounding phenolic molecules such as ferulic acid residues typically found in plant arabinoxylan.^[^ ^216^, ^223^, ^225^ ^]^

Covalent bonds in food gels often improve the structural qualities of the gel in terms of resilience and rigidity. Soy protein gels prepared with TGase displayed strong, true gel characteristics in rheology as compared to salt‐ and acid‐induced gels with an *n* value of 0.0167 compared to 0.1027 and 0.0944. Moreover, the texture of the gel was reported to be strongly correlated to Glu and Lys fractions and their ratios due to the mechanism of TGase cross‐linking.^[^ ^199^ ^]^ The strong chemical bonds created by enzymes had been momentous in creating an analog to the strong disulfide bond network created by gluten in bread. Gluten‐free dough supplemented with brown rice, white rice, and quinoa flour and crosslinked by TGase (0.5–2U g^−1^) saw a reduction hardness and an increase in springiness and gas holding capacity.^[^ ^226^ ^]^ The enhancing effects were not seen in samples where oat flour was added.^[^ ^226^ ^]^ Tyrosinase and xylanase may be used to treat oat‐based bread to lower hardness and an increase in the specific volume of the loaf.^[^ ^227^ ^]^ Enzymatic cross‐linked protein often yields desirable texture quality, however TGase‐induced gelation of soy protein gels may result in lower digestibility due to the formation of isopeptide bonds.^[^ ^228^ ^]^

Disulfide bonds are formed between the cysteine residues between or within chains of protein. Figure 8b shows a S‐S bridge formed through the oxidation of the thiol groups (R‐S‐H) between two cysteine residues, creating a covalent linkage between or within protein chains.^[^ ^233^, ^234^ ^]^ Cysteine residue, located in certain plant proteins such as soy 11S glycinin,^[^ ^235^ ^]^ zein,^[^ ^236^ ^]^ and gluten,^[^ ^237^ ^]^ contributes to the gelation properties of their respective gels. The formation of an elastic gel structure by wheat gluten is a prime example of this. Cysteine residues within glutenin (low and high molecular weight) and ɑ‐ (six residues), γ‐gliadins (eight residues) were allowed to form both inter‐ and intra‐molecular S‐S bonds during rehydration and mixing of the flour with water.^[^ ^238^, ^239^ ^]^ In particular, polymerized glutenins were believed to be responsible for the gel's elasticity.^[^ ^237^ ^]^ As a result, larger protein aggregates were further formed through covalently bonded glutenin subunits of all molecular weights. These aggregates further interact via intermolecular bonds to form an elastic and extensible gel.^[^ ^237^, ^240^ ^]^ Further processing and additives to native plant protein have also proven successful in increasing free sulfhydryl groups and disulfide bonds. High‐intensity ultrasonic pre‐treatment of soy protein was shown to increase disulfide bonds in CaSO_4_‐induced soy protein gel, resulting in a substantial increase in WHC and gel strength.^[^ ^241^ ^]^ Moreover, the incorporation of L‐cysteine into mung bean protein isolate and wheat gluten was also shown to increase intermolecular disulfide bonds.^[^ ^242^ ^]^ Adding 0.4% L‐cysteine was responsible for a 17.59× increase in the hardness of the gel. As seen in SEM images, the addition of 0.4% L‐cysteine was shown to create a denser network, along with detrimental effects seen in the excessive addition of 0.6% L‐cysteine. The result indicates that the appropriate addition of L‐cysteine enhanced the mechanical properties of structures bound by disulfide bonds. The development of this technique shows the potential of using protein molecules as the main structure to replace polysaccharides in firm texturized food.

#### 3.3.2 Identification and Quantitative Observation of Sol‐Gel Transition

As implicated in Section 3.3.1, phase change between sol‐gel is dependent on the type of protein/polysaccharide and or the involvement of a coagulant. Likewise, the observation of phase change would also have to be adapted depending on the system. The two methods that are largely employed by researchers revolve around observing the change in rheology over temperature or time and differential scanning calorimetry (DSC).

Oscillatory rheology is an important tool used by researchers to understand the viscoelasticity at each temperature or point in time. Since a gel is defined by the difference in its moduli (G′ > G″), the crossover point between the two moduli indicates the transition between a predominantly viscous system (sol) and a predominantly elastic system (gel).^[^ ^243^ ^]^ For temperature‐dependent gel systems, temperature sweep in oscillatory rheology is effective in identifying the gelation temperature during both heating and cooling.^[^ ^243^ ^]^ Temperature sweep is also effective in showing thermal irreversibility in gels such as that of konjac glucomannan^[^ ^244^ ^]^ and CUD,^[^ ^245^ ^]^ whereupon reheating of a previously heat‐set gel, cross‐over between the moduli will not reoccur. Furthermore, a time sweep is ideal in systems where gelation is gradual or delayed. This is shown by Yang et al.^[^ ^209^ ^]^ where gelation of soy protein by lactic acid fermentation and GDL was observed after 74 and 55 min respectively following incorporation.

**Figure 9:**
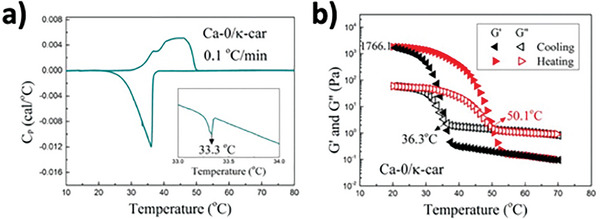
DSC and temperature sweep in dynamic rheology of κ‐carrageenan gel. a) DSC thermograms of 2%(w/w) κ‐carrageenan hydrogel at 0.1 °C min^−1^ cooling and heating rate. b) Temperature sweep in dynamic rheology analysis at a controlled frequency (1 rad s^−1^) and strain (1%) showing dependency of storage and loss moduli (G′ and G″). Adapted with permission.^[^ ^250^ ^]^ Copyright 2016, Elsevier.

Given the change of state is a thermal event, the sol‐gel transition temperature (T_SG_) may be observed by DSC.^[^ ^191^ ^]^ Gelation may be either an endothermic or exothermic event, depending on the gelling agent. For instance, an exothermic peak was observed for the gelation of κ‐carrageenan (cold‐set), while endothermic peaks were observed by heat‐induced gelation of pea protein.^[^ ^246^, ^247^ ^]^ As such, T_SG_ is identified by the maximum of the endothermic/exothermic peaks. Multiple peaks were also observed in some systems corresponding to different thermal events in the gelation process. Heat‐induced gelation of proteins was observed to contain 2 peaks in DSC thermographs corresponding to the initial denaturation of the protein (lower temperature) as well as the gelation of protein aggregates (higher temperature).^[^ ^248^, ^249^ ^]^ In studies where both DSC and time/temperature sweep in rheology were conducted, consistency was observed between the two methods^[^ ^250^, ^251^ ^]^ (**Figure** **9**).

#### 3.3.3 Roles of Protein Gel in Food

The abundance of roles plant material may play makes engineering solutions to solve environmental, health, and medical challenges feasible. Food gels are often created as hydrogels where the gel matrix exists within the aqueous phase. This includes traditional tofu, konjac jelly, and fruit preserves. Nonetheless, emerging research in food hydrogel focuses on the texturization of plant‐based muscle‐like meat mimetics^[^ ^242^, ^252^ ^]^ and delivery vehicles for active ingredients.^[^ ^253^, ^254^ ^]^

Emulsion gels may be created through inducing gelation with the aqueous phase in an oil‐in‐water (O/W) emulsion.^[^ ^255^ ^]^ Emulsion‐filled protein gels have been considered as a way to optimize food texture.^[^ ^256^ ^]^ These are soft solid‐like materials consisting of a protein gel containing dispersed oil‐in‐water emulsion droplets.^[^ ^257^ ^]^ This schematic allows for the creation of various types of emulsion‐based food such as dairy (cheese,^[^ ^258^, ^259^, ^260^ ^]^ yogurt,^[^ ^261^, ^262^ ^]^ condiments (mayonnaise^[^ ^263^, ^264^ ^]^), and fat replacers.^[^ ^244^, ^265^, ^266^, ^267^ ^]^ Both protein and polysaccharides may be used as an emulsifier or gelling agent in this arrangement.^[^ ^268^ ^]^

Food gels were also created as a carrier of nutrients and bioactive ingredients. Food gels were proven to be able to protect active ingredients from environmental stressors, including UV, oxidation, and temperature.^[^ ^269^, ^270^ ^]^ The release profile may also be modulated by the gel matrix to achieve a specific release profile.^[^ ^271^ ^]^ Soft gels are also a safe way to deliver water and nutrients to dysphagia patients.^[^ ^272^ ^]^

## 4 Characterization of Plant Proteins as Food Materials

### 4.1 Structural Properties

Plant proteins are more hydrophobic, prone to aggregate, and inflexible compared to animal proteins, and thus modification of plant proteins is essential to bring their functionality closer to animal proteins.^[^ ^10^ ^]^ Physical, chemical, and biological processing significantly alter the molecular interaction and structure of plant proteins, influencing their functionality as extensively reviewed.^[^ ^17^, ^107^, ^273^, ^274^ ^]^ Therefore, the focus of this section is to discuss quaternary, tertiary, and secondary structures of plant proteins (**Table** **1**).

Protein quaternary structure is a cluster of polypeptide chains, indicating protein fractions. The quaternary structure of plant proteins is readily destroyed by typical processing including, heat, alkali, high pressure homogenization, glycation, etc.^[^ ^17^, ^275^, ^276^ ^]^ Methods for characterizing protein quaternary structure typically include size exclusion chromatography, gel electrophoresis, and X‐ray crystallography.^[^ ^97^, ^99^, ^277^ ^]^ In many cases, the plant protein aggregates are broken down into smaller units with physical processing.^[^ ^93^, ^97^ ^]^ For example, high‐pressure processed soy protein isolate characterized by native‐polyacrylamide gel electrophoresis (PAGE) showed a band at >250 kDa, but no bands in smaller molecular weights, indicating high pressure processing only broke down the protein aggregates, and no monomeric subunits were generated.^[^ ^93^ ^]^ However, with pH shift, a common chemical treatment, individual monomers could be obtained in a molten state due to charge repulsion from exposure to extreme pH conditions.^[^ ^278^ ^]^

Tertiary structure refers to the degree of protein folding and determines surface hydrophobicity, which is related to the distribution of hydrophobic amino acids between the interior and exterior of a folded peptide chain mostly driven by hydrophobic interaction.^[^ ^10^ ^]^ Although many studies have characterized the degree of protein unfolding upon modification using fluorescence spectroscopy, the impact of different treatments on the tertiary structure has not been fully established. Most studies only reported to the extent that loss of tertiary structure or protein unfolding has occurred.^[^ ^107^, ^273^, ^276^, ^279^, ^280^ ^]^ The tertiary structure, referring to a 3D assembly of the polypeptide chains under various mechanisms in their interactions via hydrogen bonding, ionic bond, and hydrophobic interaction, is generally analyzed by fluorescence spectroscopy, UV‐Vis spectroscopy, nuclear magnetic resonance spectroscopy (NMR), etc., providing details on morphology, crystallinity, and chemical composition, which cannot be simply explicable. More investigation on how the tertiary structure might change upon processing is needed to thoroughly understand protein unfolding.

Secondary structures such as α‐helix, β‐sheet, β‐turn, and random coil, are influenced largely by hydrogen bonding and play an important role in protein folding and forming food structure. Plant proteins mainly consist of β‐sheet, β‐strands, and β‐turn, while α‐helix and random coil are present in relatively low proportion.^[^ ^281^ ^]^ The secondary structure of plant proteins is highly dependent on pH.^[^ ^281^ ^]^ Peanut protein, for example, shows an increase in α‐helix and a decrease in β‐sheet at pH 2, while a decrease in α‐helix and an increase in β‐sheet were found at pH 10 or higher, indicating more ordered structures exist in acidic conditions and protein unfolding and dissociation in high alkaline condition.^[^ ^278^, ^282^ ^]^ Quantification of protein secondary structure is commonly done by Fourier transform infrared spectroscopy, Raman spectroscopy, and circular dichroism. Recently, microfluidic modulation spectroscopy (MMS) using automated infrared technology was introduced as a reliable method for evaluating the protein secondary structure in biotherapeutic formulations with a wide range of concentrations covered. Food protein complexes characterized by using MMS demonstrated reproducible data with >99.5% repeatability and its advantage of measuring original samples directly without significant dilution as required in circular dichroism, thus showing significant discrepancies in relative secondary structure contents to circular dichroism.^[^ ^283^ ^]^

Modification of plant proteins significantly impacts secondary structure toward more flexible and irregular structure from highly ordered structure. With Maillard reaction, a tendency of a decrease in α‐helix and an increase in random coil suggested the destruction of ordered structure due to the heat used to covalently bind proteins with polysaccharides.^[^ ^284^ ^]^ High pressure homogenization and pH shifting to 12 also showed a decrease in α‐helix and an increase in random coil, indicating a looser and more irregular form from a rigid structure.^[^ ^285^ ^]^ Interestingly, in the case of ultrasound treatment, the degree of change in the secondary structure depends on the intensity and sonication time.^[^ ^92^ ^]^ In ultrasound‐treated soy protein, α‐helix and random coil decreased and β‐sheet increased at low intensity (200 W), while α‐helix increased, and β‐sheet decreased.^[^ ^286^ ^]^ This may be related to partial unfolding of plant proteins and cluster formation during ultrasound treatment as mentioned previously (Figure 2b).

Protein conformation and dynamics in their structure could be identified by solid‐state NMR for membrane protein structure and ligand binding,^[^ ^287^ ^]^ differential scanning calorimetry for molecular mobility and crystallization kinetics,^[^ ^288^ ^]^ and X‐ray diffraction for powder structure (amorphous/crystalline).^[^ ^289^ ^]^ The state‐of‐the‐art technologies for protein quantification, typically developed in pharmaceutical industry and medical research, are encouraged to broaden their applications to food protein analysis for robust characterization of protein structure in complex food formulations. Moreover, protein analytical methods using light sources such as neutron, X‐ray, and infrared radiation are emerging. For example, small angle X‐ray scattering (SAXS) could indicate the structure of sodium caseinate in solution, whereas the structure of sodium caseinate attached to the oil/water interface could be estimated by small angle neutron scattering.^[^ ^290^ ^]^ BioSAXS has been recently applied for radiation‐sensitive materials or proteins with low concentration to quantify their structure in chemical and biological systems.^[^ ^291^, ^292^ ^]^ Synchrotron‐FTIR technique has been extensively applied in food samples with various structure including powder,^[^ ^156^ ^]^ gel,^[^ ^293^ ^]^ and emulsions^[^ ^294^ ^]^ for detecting the location of protein, polysaccharide, and lipid by creating chemical maps based on FTIR spectra obtained in a particular area.^[^ ^295^, ^296^ ^]^ Synchrotron radiation circular dichroism is also a popular method to analyze protein secondary at oil/water interface.^[^ ^297^ ^]^ Nevertheless, the application of these technologies is needed to quantify newly developed plant‐based samples for effective design of plant‐based food products.

**Table 1:** Characterization methods for food protein structure in various formulations.

Structure	Analytical methods	Principle	Application
Quaternary	Size exclusion chromatography	Filtration/separation by size (hydrodynamic radius)	Fractionation of zein;^[^ ^298^ ^]^ cross‐linking of pea proteins^[^ ^299^ ^]^
	Gel electrophoresis	Separation of charged protein	Isolation, purification, and identification of coconut protein^[^ ^300^ ^]^
Tertiary	UV–vis spectroscopy	Protein absorption at a certain wavenumber	Detection of protein‐ligand conjugation^[^ ^301^ ^]^
	Fluorescence spectroscopy	Emission of residual aromatic amino acids	Component analysis;^[^ ^302^ ^]^ detection of binding protein with other ligands^[^ ^303^ ^]^
Secondary	FTIR, raman, NIR	C = O, N‐H, C‐N vibrations	Secondary structural changes of food proteins upon processing/in complex food^[^ ^304^, ^305^, ^306^, ^307^ ^]^
	Circular dichroism	Unequal absorption of left‐handed and right‐handed circularly polarized light	
	Microfluidic modulation spectroscopy	a tunable quantum cascade laser mid‐infrared detecting mid‐infrared region	Secondary structural characterization of mixed food protein complexes^[^ ^283^ ^]^
Conformation	Nuclear magnetic resonance	^1^H, ^13^C, ^15^N chemical shifts	Molecular structure and dynamics of food proteins^[^ ^308^ ^]^
	Small angle X‐ray/neutron scattering	Intensities of X‐rays/neutron scattered by a sample as a function of scattering angle	Structure of sodium caseinate in dispersions/at oil/water interface;^[^ ^290^ ^]^ protein structural changes during digestion^[^ ^309^ ^]^
	Synchrotron‐FTIR	Employing the intense beam produced by a synchrotron as its light source	Lipid/protein distribution in food powder,^[^ ^156^ ^]^ Mozzarella cheese,^[^ ^310^ ^]^ rice grain^[^ ^311^ ^]^
	Synchrotron radiation circular dichroism		Protein secondary structure at oil/water interface^[^ ^297^, ^312^ ^]^

### 4.2 Morphological Properties

**Figure 10:**
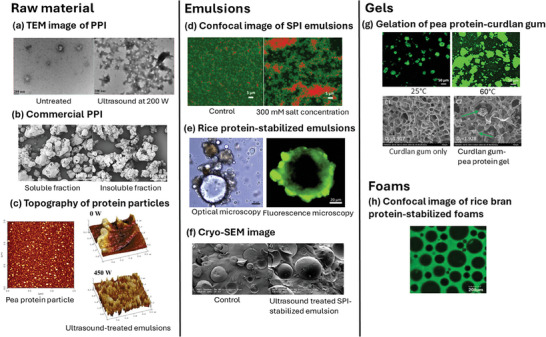
Morphology of plant proteins in various food formulations. a) Transmission electron microscopy (TEM) image of PPI (Adapted with permission.^[^ ^99^ ^]^ Copyright 2022, Elsevier and Copyright 2022, Elsevier). b) Commercial PPI by scanning electron microscopy (SEM) (Adapted with permission.^[^ ^96^ ^]^ Copyright 2022, Elsevier). c) Plant protein particles by atomic force microscopy (AFM) (Adapted under the terms of the CC‐BY license.^[^ ^315^, ^316^ ^]^ Copyright 2024, Elsevier and Copyright 2022, Elsevier). d) SPI‐stabilized emulsions by confocal laser scanning microscopy (CLSM) (Adapted with permission.^[^ ^114^ ^]^ Copyright 2019, Elsevier). e) Rice protein‐stabilized emulsions by optical and fluorescence microscopy (Adapted with permission.^[^ ^319^ ^]^ Copyright 2021, Elsevier). f) Plant protein‐stabilized emulsions by cryo‐SEM (Adapted with permission.^[^ ^100^ ^]^ Copyright 2016, Elsevier). g) Gelation of pea protein‐curdlan gum by CLSM and SEM (Adapted with permission.^[^ ^180^ ^]^ Copyright 2023, Elsevier). h) Rice protein protein‐stabilized foams by CLSM (Adapted with permission.^[^ ^322^ ^]^ Copyright 2022, Elsevier). PPI: pea protein isolate, SPI: soy protein isolate.

Morphology of plant proteins in the form of solutions, emulsions, foams, gels, and powders is crucial to understanding protein structure and functions in food formulations. Transmission electron microscopy (TEM) and scanning electron microscopy (SEM) with lyophilized samples are often used to characterize protein shape and structure in solutions and suspensions. Commercial pea protein isolates have large aggregates when using TEM, while a coarse network is observed with high‐intensity ultrasound treatment (**Figure** **10**a).^[^ ^99^ ^]^ For SEM, spherical particles of untreated PPI are seen (Figure 10b).^[^ ^96^, ^97^ ^]^ The smoothness and surface porosity could be observed by SEM, which allows visual distinction between samples according to physicochemical treatment.^[^ ^97^, ^277^, ^313^ ^]^ In addition, high solubility is expected with a smooth surface of a protein.^[^ ^60^, ^313^ ^]^ For example, salt‐extracted hemp protein shows a homogeneous and smooth surface compared to alkaline extraction‐isoelectric precipitation, resulting in higher solubility between pH 2–7.^[^ ^60^ ^]^ Moreover, after extraction, freeze drying forms larger protein particles with breadcrumb‐like particle shapes, while spray drying forms spherical particles.^[^ ^77^, ^314^ ^]^

Atomic force microscopy (AFM) is also widely used to observe topography such as protein aggregation, particle, and droplet aggregation. In particular, AFM shows the height of the samples, making it possible to estimate the size of the protein oligomer and cluster (Figure 10c). Soy protein aggregation upon ultrasound was observed subject to intensity suggesting that increasing ultrasound intensity leads to uniform particle formation alleviating particle aggregation to some extent (up to 450 W in this study), whereas a tendency of protein aggregation was found with excessive ultrasound (600 W).^[^ ^315^ ^]^ Moreover, AFM analysis was used to investigate the interfacial structure of pea protein aggregates, showing extra thin interfacial film (0–1 nm) formation from different pea protein fractions despite the presence of large insoluble protein aggregates (>1 µm).^[^ ^316^ ^]^

In the case of emulsion, droplet size, flocculation, and coalescence can be observed by optical microscopy, while confocal laser scanning microscopy (CLSM) is useful to observe the location of oil droplets and proteins. In most cases, a thick layer of protein surrounds the oil droplet (Figure 10e).^[^ ^114^, ^317^, ^318^ ^]^ The structure of the protein interfacial layer was observed in SPI‐stabilized emulsions according to salt concentration and protein aggregates surrounding oil droplets could be observed (Figure 10d).^[^ ^114^, ^319^ ^]^ CLSM is also used to characterize the structure of foams to estimate the bubble size and size changes after foam formation (Figure 10h).^[^ ^314^, ^320^ ^]^ Cryo‐SEM is a useful tool to characterize oil droplets and interfacial layers. The position of proteins and how the protein surrounds the surface of droplets of emulsion stabilized by soy protein were observed using cryo‐SEM, suggesting that soy protein before ultrasound treatment formed an interfacial layer with a bumpy and textured layer, while smoother interfacial layer was formed in the ultrasound‐treated soy protein emulsion (Figure 10f).^[^ ^100^, ^318^ ^]^

In the case of gel, SEM is most often used to observe the size of aggregates, degree of cross‐linking, and pore size on the surface and cross‐section.^[^ ^60^, ^61^ ^]^ A honeycomb structure is observed in protein gels and the homogeneity of the gel could be decided upon protein treatment before gelation. Furthermore, CLSM and SEM could be used together to understand the gelation behavior.^[^ ^180^, ^321^ ^]^ Sol‐gel transition according to temperature was observed using CLSM in PPI‐curdlan gum gelation and their network of protein and polysaccharide was observed by SEM (Figure 10g).

### 4.3 Rheological Properties

**Figure 11:**
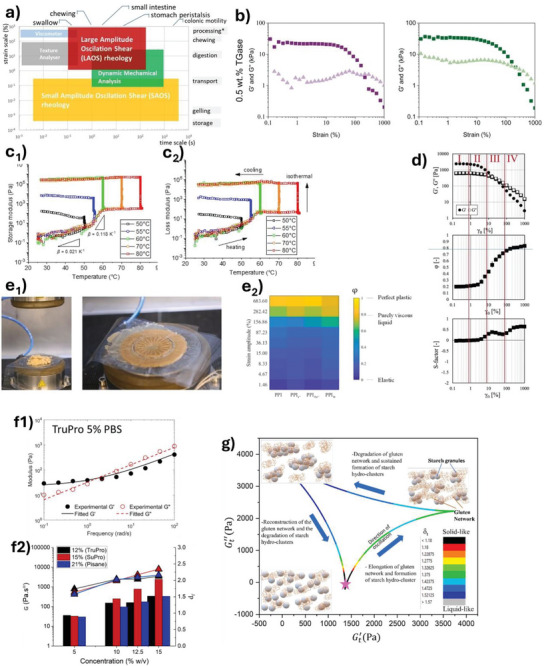
Rheological methodology and properties of plant proteins. a) illustration of different strain/time scales in rheology measurements to represent typical food processing and digestion conditions. b) Storage and loss modulus of pea protein isolate (left) and mung bean protein isolate (right) with 0.5 wt.% transglutaminase (TGase) concentrations as a function of strain amplitude at a frequency of 1 Hz at 120 °C (Reproduced with permission.^[^ ^327^ ^]^ Copyright 2023, Elsevier). c1) storage modulus & c2) loss modulus during the gelling process of pea protein isolate with curdlan gum at different heating and cooling patterns (Reproduced with permission.^[^ ^180^ ^]^ Copyright 2023, Elsevier). d) Large amplitude oscillation shear (LAOS) sweeps of fermentation‐induced pea protein gels made from pea protein pre‐treated at pH 7.0 at intercycle strain amplitudes γ0 between 0.1% and 1010% (Reproduced with permission.^[^ ^321^ ^]^ Copyright 2020, Elsevier). e1) before (left) and after (right) a rheological measurement in a closed cavity rheometer; e2) dissipation ratio maps of pea protein isolate/carbohydrate blends (93:7) at 30 °C and heated at 140 °C and cooled to 30 °C (Reproduced with permission.^[^ ^328^ ^]^ Copyright 2022, Elsevier). f1) FKVM model fitting of storage and loss moduli for pea protein isolates gels at 5% (w/v), and f2) the effect of protein concentration and solubility on quasi‐property and fractal dimension of pea protein gel (Reproduced with permission.^[^ ^332^ ^]^ Copyright 2024, Elsevier). g) Sequence of physical processes (SPP) analysis of gluten network and starch hydro‐clusters under LAOS, showing structural changes during oscillation (Reproduced with permission.^[^ ^335^ ^]^ Copyright 2022, Springer).

Rheology serves as a crucial tool for understanding the textural and structural transformations of plant‐based protein systems, encompassing both the overall behavior through bulk rheology and the interactions at various interfaces via interfacial rheology.^[^ ^256^ ^]^ Rheology demonstrates the viscosity and modulus of plant proteins under various processing conditions such as mixing, transport, gelation, extrusion, and storage, allowing for analysis across different strain scales.^[^ ^256^ ^]^ This also extends to the digestive process of plant proteins, including chewing, swallowing, and the peristalsis of the stomach and intestines,^[^ ^323^ ^]^ manifesting over varying temporal scales (**Figure** **11**a). Meanwhile, the mechanical properties of food reflect the structural integrity of bulk food items.^[^ ^324^ ^]^ Together, these characteristics provide a multidimensional representation of how plant‐based protein foods respond to stress and deformation, offering critical stress and structural feedback across different scenarios.

#### 4.3.1 Bulk Rheology of Plant Proteins

The rheological behavior of plant proteins in solutions is instrumental for understanding their functional performance in food systems, with viscosity measurements revealing how these proteins interact in liquid environments. Prediction of the behavior in processes like mixing, dilution, and particularly in complex systems is feasible where plant proteins often present as multi‐component solutions, suspensions, or emulsions.^[^ ^325^ ^]^ As mentioned earlier, the stability of plant protein suspensions/emulsions is crucial for subsequent processing steps,^[^ ^326^ ^]^ where a higher viscosity tends to favor the stratification of fine particles in suspensions and helps in the separation of dispersed phase droplets in emulsions to prevent coalescence.^[^ ^256^ ^]^ For example, after the addition of a small amount of transglutaminase, both pea protein and mung bean protein can form gel‐like structures, indicated by an increased G′ (Figure 11b).^[^ ^327^ ^]^ However, overly viscous systems can negatively impact the further processing of plant proteins, posing challenges in homogenization or during spray drying for microencapsulation, making it difficult to achieve the desired product characteristics and efficiency.^[^ ^328^ ^]^

Gelation of plant protein involves the transition from a liquid to a gel state, influencing the textural properties.^[^ ^329^ ^]^ Understanding gelling behavior using rheological characterizations helps to create networks capable of water retention and providing firmness, relevant in plant‐based yogurts, desserts, and meat analogs, where gel strength and elasticity are key.^[^ ^256^ ^]^ The storage and loss modulus of the pea protein – curdlan gum mixture could demonstrate the gel formation process during both the heating and cooling stages, illustrating the two different gelling mechanisms (thermal‐reversible and thermal‐irreversible) within the same system (Figure 11c).^[^ ^180^ ^]^ The transition of pea protein gels from elasticity to plasticity is demonstrated by the transformation of the Lissajous curve from a bone‐like to a rectangular shape. Additionally, the shift in the strain‐stiffening factor from negative to positive offers further confirmation of this behavior, as depicted in Figure 11d.^[^ ^321^ ^]^

During extrusion, plant proteins undergo textural transformations under high temperature and shear. Elasticity, viscosity, and melt strength become critical, in determining the formation of fibrous, meat‐like textures.^[^ ^330^ ^]^ The closed cavity rheometer (Figure 11e1) could monitor the structure formation by using the storage/loss modulus to mimic the heat and pressure during extrusion (Figure 11e2).^[^ ^328^ ^]^ Understanding these properties is essential for optimizing extrusion parameters to achieve desired textures in plant‐based meat alternatives, aiming to replicate the chewiness and fibrous nature of animal meat.^[^ ^331^ ^]^

Figure 11f1,f2 introduce a novel rheological methodology utilizing the Fractional Kelvin‐Voigt Model (FKVM) to characterize plant‐based food gels.^[^ ^332^ ^]^ Figure 11f1 shows the FKVM model fitting of the storage and loss moduli for pea protein isolates in phosphate‐buffered saline (PBS) at various concentrations. This innovative approach captures the viscoelastic response across a wide frequency range, providing insights into the gelation behavior and network strength of the proteins. Figure 11f2 illustrates the effect of protein concentration and solubility on the quasi‐property *G* and the fractal dimension *d_f_* of the protein gels.^[^ ^333^ ^]^ This model not only introduces the quasi‐property as a parameter but also includes a fractional description of the gel structure, enabling a detailed analysis of the gelation mechanisms.^[^ ^332^ ^]^ The FKVM showed great potential for broader applications beyond pea and soy proteins, expanding the characterization capabilities for various plant‐based food systems.

Lee and Rogers^[^ ^334^ ^]^ introduces a novel analytical method known as the Sequence of Physical Processes (SPP) to interpret Large Amplitude Oscillatory Shear (LAOS) data. This method provides a comprehensive framework for understanding the complex viscoelastic behavior of plant‐based food gels under dynamic conditions. Figure 11g demonstrates the application of the SPP methodology to gluten network and starch hydro‐clusters in food systems.^[^ ^335^ ^]^ This analysis reveals the intricate structural changes that occur during the oscillatory process, offering deeper insights into the formation and degradation mechanisms of the food matrix. The SPP method thus extends our capability to analyze and optimize the texture and stability of plant‐based foods by providing a detailed understanding of their microstructural dynamics.^[^ ^334^ ^]^ The application of SPP in food systems has been summarized in a recent review.^[^ ^336^ ^]^

#### 4.3.2 Interfacial Rheology of Plant Proteins

Interfacial rheology plays a pivotal role in delineating the formation and stabilization mechanisms of emulsions, offering insights beyond conventional analysis by examining interactions at the interface.^[^ ^337^ ^]^ This approach, incorporating techniques like large amplitude oscillatory shear (LAOS) analysis through Lissajous curves and Chebyshev decomposition, elucidates the complex behaviors of plant‐based proteins under various conditions such as changes in fat volume, emulsifier ratios, pH levels, and homogenization pressures.^[^ ^256^ ^]^ Studies, including pea protein's efficacy in Pickering emulsions,^[^ ^338^ ^]^ and soy proteins’ displacement by Tween 20 in oil‐in‐water emulsions,^[^ ^339^ ^]^ highlight the method's ability to predict emulsion stability and improve understanding of protein‐emulsifier interactions.

## 5 Food Applications

### 5.1 Plant‐Based Meat Analogs

The animal meat and dairy industry produces 60% of total emissions arising from the agricultural sector.^[^ ^340^ ^]^ In 2019, the EAT‐Lancet Commission^[^ ^341^ ^]^ presented the global consensus on a healthy diet related to sustainability and climate change and recommended caloric intake from peas and beans (172 kcal day^−1^) should be 5 times more than that from red meat (30 kcal day^−1^) till 2050. Plant‐based meat analogs represent a burgeoning field aiming to replicate the sensory and nutritional qualities of meat using plant‐derived ingredients. Key to the development of these products is the utilization of texturization techniques such as extrusion, which aligns plant proteins in a fibrous structure reminiscent of muscle tissue.

Advances in extrusion technology have enabled the creation of highly textured plant‐based proteins that closely resemble the mouthfeel of meat. Through controlled cooking, cooling, and shearing processes, plant proteins are restructured to form the fibrous textures necessary for meat analogs, using proteins from peas, wheat, fava beans, chickpeas, mung beans, and many more.^[^ ^331^, ^342^, ^343^, ^344^, ^345^ ^]^ While emulsion destabilization occurs during extrusion, leading to the loss of fat and fat‐soluble nutrients from the protein network, the marbling and juiciness of meat from fat can still be recovered by incorporating plant‐based fats and binders.^[^ ^346^, ^347^ ^]^ Natural fats from sources like coconut oil and shea butter, along with plant‐based binders such as methylcellulose, are used to achieve the desired fat distribution and texture.^[^ ^348^ ^]^

Single‐screw and twin‐screw extrusion are the principal technological methods for texturizing plant proteins. The necessity of employing extrusion technology primarily arises from the challenge posed by the abundance of globular proteins in plant sources, which do not readily form the fibrous structures characteristic of animal proteins.^[^ ^349^ ^]^ Such fibrous structures are crucial for replicating the unique textures and sensory experiences of meat and meat products. Extrusion processes, leveraging short‐duration high‐temperature and high pressure treatments, are capable of unfolding the intact structure of globular proteins, leading to the formation of interconnected networks.^[^ ^350^ ^]^ This transformation is instrumental in mimicking the structure of meat proteins, representing one of the key technological advancements in the industrialization of plant‐based meat alternatives in recent years.

Extrusion technology has found successful application across a variety of plant proteins, with its earliest use being in soy protein, where it facilitated the formation of a cross‐linked network structure in the textured soy protein.^[^ ^351^ ^]^ Despite the decades‐old history of this technology, the recent developments are primarily characterized by a shift from traditional low‐moisture extrusion techniques to novel high‐moisture extrusion technologies.^[^ ^352^ ^]^ High‐moisture extrusion is distinguished by its ability to foster the formation of fibrous structures, which exhibit enhanced water and oil retention capabilities.^[^ ^353^ ^]^ Moreover, recent studies have revealed that high‐moisture extrusion enables soy protein to form an anisotropic structure at material temperatures of 124–135 °C, characterized by a water‐rich dispersed phase enveloped by a continuous, water‐poor, or protein‐rich phase.^[^ ^354^ ^]^ One way to further enhance the formation of fibrous structures in plant proteins is by incorporating additional components. Adding 6% sodium alginate into soy protein significantly benefits the fibrous structure formation in extrudates by promoting high rehydration and protein digestion rates. These advantages are attributed to disulfide bonds and hydrogen interactions in developing fibrous structures, alongside the transformation of protein secondary structures from α‐helix to β‐sheet content.^[^ ^343^ ^]^ Furthermore, incorporating 10 wt.% soy protein fibrils (from freeze‐dried soy protein) led to a 35% reduction in the hardness of soy protein isolate extrudates and a 32% increase in their anisotropic index, significantly enhancing water holding capacity and cooking stability while reducing bitterness and astringency by 46% and 33%, respectively.^[^ ^355^ ^]^

While soy protein exhibits superior material characteristics, its beany flavor and allergenicity have, in recent years, shifted research focus toward the extrusion puffing of other plant proteins.^[^ ^356^ ^]^ In the extrusion process, pea protein undergoes structural changes primarily through the unfolding of α‐helix structures to form β‐sheet and β‐turn structures, a transformation that plays a pivotal role in developing its anisotropic fibrous texture.^[^ ^357^ ^]^ Unlike soy protein, pea protein relies more on the addition of other components to enhance its textural integrity, with elements such as amylose/amylopectin,^[^ ^342^ ^]^ L‐cysteine,^[^ ^358^ ^]^ fatty acids,^[^ ^359^ ^]^ and various polysaccharides^[^ ^344^ ^]^ playing crucial roles in improving the texturized pea protein's texture. These additives contribute to a tighter linkage within the protein matrix, facilitating the development of a more desirable and meat‐like fibrous structure.^[^ ^356^ ^]^ Following pea protein, other types of plant proteins that can undergo texturization through extrusion technology include wheat gluten,^[^ ^360^ ^]^ chickpea protein,^[^ ^361^ ^]^ fava bean protein,^[^ ^345^ ^]^ and mung bean protein,^[^ ^331^ ^]^ among others. These proteins, when subjected to extrusion, can similarly benefit from the process's ability to alter protein structure^[^ ^362^ ^]^ and enhance textural properties.^[^ ^353^ ^]^

### 5.2 Plant‐Based Dairy Analogues

Plant‐based emulsions and gels have been investigated as a replacement for dairy products. Dairy milk is a suspension of dispersed milk fat stabilized by casein with dissolved lactose, minerals, vitamins, and whey protein.^[^ ^363^ ^]^ As plant materials could develop analogous roles occupied by milk proteins in the form of an emulsion or emulsion gel, many researchers attempted to recreate key properties of dairy products using an array of processing techniques and formulations.

#### 5.2.1 Milk/Cream

Current commercial plant‐based milk analogs largely exist as physically and thermally extracted plant materials with the possible addition of additives to modify the stability and sensory quality.^[^ ^364^ ^]^ Challenges faced by current plant‐based milk include an incomplete nutrition profile, off‐flavors, and instability.^[^ ^364^ ^]^ Thus, additional processing of plant‐based ingredients was investigated to improve these qualities of plant‐based milk analogs.^[^ ^364^ ^]^ Sedimentation of insoluble plant components and instability of the dispersed phase limits the applicability and acceptability of plant‐based milk.^[^ ^364^ ^]^

To improve the solubility and emulsifying properties of proteins, enzymatic treatment of native plant proteins was investigated. Alcalase treatment of up to 16% degree of hydrolysis was found to increase the solubility of fava protein in water and hence emulsion stability against flocculation, coalescence, and creaming.^[^ ^365^ ^]^ Improvement of intermediate degree of enzyme hydrolysis was also reported in cashew nut milk. It was observed that 60 min of bromelain (600 AU g^−1^) achieved higher sedimentation and creaming stability than cashew milk, which was treated with a higher amount of bromelain or over a longer period.^[^ ^366^ ^]^ In each of the cases, enzymatic hydrolysis was reported to increase ζ‐potential and decrease the surface hydrophobicity of the emulsion, enhancing the stability.^[^ ^365^, ^366^ ^]^ A combined use of plant polysaccharides and proteins has also been investigated to create a stable emulsion. For instance, gum Arabic was found to improve the stability of both flaxseed and soybean protein emulsions through an increase in viscosity and competitive surface adsorption, respectively.^[^ ^367^ ^]^ Gum Arabic and Xanthan gum were found to improve the stability of a double emulsion (water‐in‐oil‐in‐water) with encapsulated amino acid.^[^ ^368^ ^]^ Such a regime may provide opportunities for nutritional fortification in plant‐based milk.

#### 5.2.2 Cheese

Several qualities that are essential for a successful plant‐based cheese analog include meltability, stretchability, texture, and aroma.^[^ ^9^ ^]^ Both the melting and stretching characteristics of a cheese analog are critical for its incorporation into certain food items such as pizzas. Commercial starch‐based cheese analogs were reported to have minimal melt spread due to the dominance of the gelatinized starch network.^[^ ^369^, ^370^ ^]^ As such, novel research pivoted to explore the use of different structural components or regimes to improve these key qualities.

Prolamin‐rich proteins were found to exhibit comparable melting and stretching quality to dairy cheese. Zein proteins were tested as the main structural component of cheddar and mozzarella analogs.^[^ ^371^ ^]^ Owing to its ability to form a flexible network when heated above its glass transition temperature, it was found to closely trace the stretching profile of cheddar cheese.^[^ ^260^, ^372^ ^]^ On the other hand, Dobson & Marangoni^[^ ^258^ ^]^ proposed a high fava protein formulation with waxy corn starch to mimic processed dairy cheese. Briefly, similarities were achieved with dairy‐processed cheese (Kraft singles) in terms of melt‐stretching when stretched vertically in the axial pull test. Although meltability was not closely comparable to dairy cheese (146.8%–184.5% of original size), their proposed formulation achieved remarkable improvement (75.8%–102%) over commercial plant‐based cheese (1.3%–24.5%).^[^ ^258^ ^]^ The study demonstrated that a network disruption system using proteins was a viable way to modulate the melting and stretching properties of cheese analogs.

#### 5.2.3 Plant‐Based Yogurt

The physical quality requirements for plant‐based yogurt analogs share similarities with both milk and cheese with some distinct differences. While off‐flavors and nutrition are challenges in plant‐based yogurt, syneresis is a distinct destabilization mechanism in these plant‐based yogurt products.^[^ ^373^ ^]^ In yogurt products, syneresis represents the separation of the serum from the bulk of the yogurt network.^[^ ^373^ ^]^ Several reasons were identified for the increase in syneresis in plant‐based yogurt, including processing parameters, formulation, bacterial culture, incorrect storage conditions, and acidification.^[^ ^373^ ^]^

Several solutions were proposed to reduce syneresis and increase the WHC of plant‐based yogurt. The inclusion of polysaccharide‐based stabilizers has been a common method to increase the stability of both dairy and plant‐based yogurt.^[^ ^374^ ^]^ For instance, tapioca starch (2%, w/w) added before the fermentation of a coconut‐based yogurt was found to reduce the syneresis rate by 3.58%–4.20% without affecting the fermentation process.^[^ ^375^ ^]^ On the other hand, the introduction of aquafaba as a gelling agent to improve stability was also reported in an oat‐based yogurt.^[^ ^376^ ^]^ In such formulation, a 13% increase in WHC and a 33% reduction in serum loss was recorded for the addition of 3% aquafaba.^[^ ^376^ ^]^ In both cases, the enhanced stability was attributed to an increase in viscosity and the formation of a gel matrix. In addition, high pressure homogenization may also improve the WHC of plant‐based yogurt. Levy et al.^[^ ^377^ ^]^ reported that high pressure treatment (0.1–200 MPa) of a potato protein‐based yogurt resulted in superior WHC (>80%) over dairy yogurt (≈40%).^[^ ^377^ ^]^ The high pressure treatment created a finer emulsion with smaller droplets that were more stable against creaming during fermentation.^[^ ^377^ ^]^ As such, the stability was believed to stem from the more homogenous acid‐induced protein gel formed during fermentation.^[^ ^377^ ^]^

## 6 Summary and Future Directions

Plant proteins are valuable resources as food ingredients. The origin of crops primarily determines the molecular characteristics and composition of plant proteins. Plant proteins display more protein fractions in a wider molecular weight range compared to animal proteins, and exist in complex aggregated forms rather than as monomers. The molecular properties of plant proteins subject to extraction and processing are critical for food structure formation, while in‐depth investigations on their extraction, processing, and functional properties in addition to sensory and nutritional properties, and food safety are needed.

Commercial plant proteins currently on the market including soy, pea, rice, wheat, oat, etc. are typically produced through alkaline extraction‐isoelectric/acid precipitation followed by spray drying in bulk forms. This process causes low water solubility due to protein denaturation, which requires additional processing steps to improve their solubility, resulting in more energy consumption and higher costs. Further technology development for plant protein extraction would be needed to produce high‐quality plant proteins. For example, the reverse micelle technique could be an alternative method for protein production, although further research is required with food grade surfactants for producing reverse micelles and evaluating the protein functionality in complex food formulations. Other emerging extraction methods including deep eutectic solvent extraction and enzyme‐assisted extraction are promising, although it is necessary to monitor protein structure change and evaluate the feasibility for bulk production.

To date, studies on utilizing plant proteins have focused on several specific legumes, cereals, and oilseeds, while the extraction and processing of other crop sources should be conducted. It would be useful to establish the relationship between extraction/processing methods and protein quality of plant sources with high protein content including lentils, chickpeas, peanuts, almonds, quinoa, chia seeds, hemp seeds, cashew, walnuts, and oats. This information could be extended when utilizing plant proteins from regional crops or reusing the residues generated from food processing.

More fundamental studies are needed to understand the functionality of plant proteins. For example, the emulsifying mechanisms of globulin‐rich legume proteins are well understood, but very few studies have addressed prolamin‐rich and/or glutelin‐rich proteins from various sources, which differ significantly in their molecular characteristics from legume proteins. The structure‐function relationship of food proteins is a topical subject, and more works on protein functionality related to molecular properties and structure are needed.

Pickering particles are expected to expand their application to emulsions and foams. Currently, only nanoparticles and/or microgel‐based Pickering emulsions are actively being studied for microencapsulation and digestion, while Pickering particles with unique structures using biopolymers and/or small molecules and/or processing technologies to tune their contact angle at oil/water interface could form a rigid interfacial layer. Pickering foams are also known to form much more stable foam than traditional plant protein‐stabilized foams, but the study on Pickering foams is in its infancy for the food industry.

Development in food gels allowed for the creation of novel gels with unique properties in texture, processing qualities, and functionality. Given the shortcomings in current plant‐based products in recreating the texture, mouthfeel, and taste of their animal‐origin counterparts, further development will have to continue exploring avenues for closer mimicry. Continued investigation of two key areas would be able to bridge the gap between the ability of plant protein and effective creation of novel food products. Further studies into various combinations of plant protein and polysaccharide systems are expected to deliver tunable characteristics to replace animal‐based products and allergenic plant ingredients. This may entail the use of regional and underutilized plant protein and polysaccharide sources. Aside from using alternate sources for protein, investigation into the interactions between different components in a plant‐based network, such as the destructive model, will allow for better accommodation of the desired properties. Furthermore, investigations in complex gel structures may also present an opportunity to improve the nutritional profile as well as the sensory attribute of plant‐based food, which will further improve the appeal of plant‐based foods. However, as the health aspect of processed food is one of the most contentious topics in the food space, consumer acceptance of the additives and processing techniques used should be considered.

## Conflict of Interest

The authors declare no conflict of interest.
